# Hæmostatic Agents

**Published:** 1856-06

**Authors:** 


### DEPARTMENT OF SELECTIONS.

### COMPARATIVE VALUE OF THE DIFFERENT HÆMOSTATIC AGENTS.

A correspondent sends us the following translation, which we publish as conveying valuable information upon an important ubject.—*Boston Med. and Surg. Jour.*

The Gazette des Hopitaux of Sept 29th, in an article on the comparative value of different substances as means of arresting hæmoptysis, after remarking that bleeding for this purpose has deservedly fallen into general disfavor, alludes to the clinical researches of Dr. Aran, published in the Bulletin Gen. de Therapeutique, and gives a *resume* of the interesting and valuable results to which he had arrived. We translate passages which seem to us of considerable value.

M. Aran has successively tried agents belonging to the class of hæmostatics, properly so called, such as resinous substances, the ergot of rye and common salt; then astringents—acetate of lead, alum *eau de Rabel*, tannin, and gallic acid; nauseants, emetics—ipecac, tartar emetic, veratrine; and sedatives of the circulation—nitre and digitalis.

Of the agents belonging to the first group, hæmostatics proper, the essence of turpentine has seemed to M. Aran especially to deserve the attention of physicians. He has prescribed it pure, in doses of from ten to thirty drops, in a glass of water, or made up into a bolus with magnesia, and taken enveloped in moistened wafer (*pain a 'chanter.*) Generally within a few hours after the patient commences taking it, there is a very marked diminution in the amount of the hemorrhage, and in twenty-four or thirty-six hours at the most, it is reduced to a very small quantity or entirely ceases. On the other hand, M. Aran is convinced, as many English and German physicians have already proved, that the essence of turpentine is less suitable in hæmoptysis, with a tendency to inflammatory action within the chest, a febrile movement, or when it occurs in young or rather plethoric subjects, than when it happens in debilitated, cachectic subjects, with characters of *passivity* or atony.

Ergot of rye and the ergotine of M. Bonjean, have shown much less efficacy against hæmoptysis than essence of turpentine. The former, even, when given in a very large dose, has seemed to exert only the most moderate influence upon the hemorrhage.

The same is not the case with chloride of sodium or common salt, which has been proved to possess an undoubted efficacy in doses of from sixty to one hundred and fifty grains taken in the course of a few hours in solution, or in the form of powder. It is particularly deserving of recommendation in such cases, as it is constantly at hand.

Among the astringents, M. Aran has found none worthy of confidence except tannin and gallic acid. Gallic acid seems to him preferable to tannin, as, with the same styptic properties, it has not the same drying action upon the tissues, and does not produce the obstinate constipation which occurs when the latter is employed. The medium dose of gallic acid, as he administered it, was from ten to twelve grains in twenty-four hours, in powders of two grains each, given at intervals of two hours.

M. Aran acknowledges the power of nauseants and emetics to arrest hæmoptysis, such as tartar emetic, ipecac and veratrine. With regard to the first two this property has been known for a long time. As for veratrine, in three cases in which it has been prescribed, the hæmoptysis was arrested as if by enchantment as soon as nausea and vomiting took place. These agents would deserve, then, to be placed in the first rank of hæmostatics, if there were not others of equal efficacy, which do not produce nausea and vomiting, effects which are always painful or disagreeable to the patient.

Nitre and digitalis have been equally, and with good reason, extolled in this case by the name of sedatives to the circulatory system. Following the example of Schmidtmann, who conceived the idea of combining sea salt with digitalis to combat hæmoptysis, M. Aran, for the same purpose, combined digitalis and nitre. This mixture, it appears, produced very remarkable results.

In ordinary cases he gave in the course of twenty-four hours four grains and a half of digitalis and twenty-three grains of nitre in four powders. But when the hemorrhage was very profuse the quantity of nitre was carried as high as thirty-eight grains, and that of digitalis to eight or even twelve grains; in some very grave cases the quantity of digitalis given was carried to twenty-three grains, and of nitre to sixty grains. A remarkable circumstance noticed was, that when these remedies were given in this quantity the system was not affected in any unfavorable manner; the pulse did not suddenly abate in frequency, nor was there a very abundant diuresis. On the other hand, the effect upon the hæmoptysis was most marked; in a few hours the flow of blood was considerably reduced, and often after twenty-four or thirty-six hours there remained only a little bloody expectoration. The diminution of the hemorrhage was generally accompanied by a great calm. Nevertheless M. Aran observed that never, after the administration of essence of nitre and digitalis, was the arrest of hemorrhage so sudden as after the administration of turpentine or gallic acid.

M. Aran sums up his opinion of the respective value of the different agents in question, in the following words: "In profuse hæmoptysis, but not immediately threatening life, the physician may take his choice of either of the preceding remedies. In very profuse hæmoptysis, on the contrary, where it is necessary to arrest the bleeding as soon as possible, and by means the least likely to depress the system, the physician cannot trust the tardy remedies. Neither the ergot, nor sugar of lead, nor *eau de Rabel*, nor alum, nor rhatany, etc., will be equal to the emergency. Only turpentine, gallic acid in a large dose, salt, nitre combined with digitalis, can be employed with success; but the necessity of proportioning the dose of the medicine to the intensity of the hemorrhage, in administering the chloride of sodium, but particularly the nitre and digitalis, is productive of great inconvenience; the danger of too great a depression from too large a dose, or from too long a continuance of the remedy.

"It is then to gallic acid and to turpentine that I give the preference in these grave cases, yet, under the apprehension of their insufficiency, I do not think the physician should limit himself to their use. It is under such circumstances that bandages applied to the limbs, which are very useful in other kinds of hemorrhage, and ice applied to the chest, have saved the life that was in danger, by stopping the hemorrhage for the moment, and allowing the internal remedies to complete the work."—*Southern Med. and Surg. Jour.*

